# Beyond the 5-Year Window: Late-Onset Ocular Morbidity and a Proposed 10-Year Functional Survivorship Protocol for Pediatric Orbital Rhabdomyosarcoma

**DOI:** 10.3390/cancers18101633

**Published:** 2026-05-19

**Authors:** Hadeel Halalsheh, Yacoub A. Yousef, Mona Mohammad, Ahmad Kh. Ibrahimi, Iyad Sultan

**Affiliations:** 1 Department of Pediatrics, King Hussein Cancer Center, Queen Rania Street, Amman 11941, Jordan; 2 Department of Pediatrics, The University of Jordan, Amman 11941, Jordan; 3 Department of Surgery (Ophthalmology), King Hussein Cancer Center, Amman 11941, Jordan; 4 Department of Radiation Oncology, King Hussein Cancer Center, Amman 11941, Jordan; 5 Artificial Intelligence Office, King Hussein Cancer Center, Amman 11941, Jordan

**Keywords:** rhabdomyosarcoma, orbit, children, outcomes, ophthalmologic late effects, MRI response, EBRT, Jordan

## Abstract

Background: Orbital rhabdomyosarcoma (RMS) is the most common primary pediatric malignant orbital tumor, typically curable with chemotherapy and radiation. Data regarding MRI chemotherapy response and long-term ophthalmologic outcomes remain limited in non-cooperative-group settings. Methods: We retrospectively reviewed children with primary orbital RMS treated at King Hussein Cancer Center (2002–2025) with vincristine, actinomycin-D, and cyclophosphamide (VAC). Pre-local-control MRI responses were classified as complete (CR), partial (PR), stable/minor (SD/MR), or progressive disease (PD). Survival and ophthalmologic outcomes were analyzed. Results: Twenty-two patients (median age 5.6 years) were included. All had localized disease (77% low-risk). All received VAC; 20 (91%) received radiotherapy (median 45 Gy). Pre-radiotherapy MRI showed 8 (36%) CR and 11 (50%) PR. Four patients (18%) died. Five-year event-free survival (EFS) and overall survival (OS) were 73% and 84%, respectively. Cataracts developed in 45% of the cohort (50% of irradiated patients) at a median of 39.1 months (range 9.4–95.1). At last assessment, visual acuity was good in 60%, moderate in 25%, and severely impaired in 15%. Conclusions: Excellent survival in orbital RMS is achievable in resource-stratified settings. Induction MRI progressive disease (PD) was associated with poor outcomes in this cohort and may represent an early prognostic signal warranting further validation in larger studies. Furthermore, the documented maximum cataract latency of 95 months suggests that the standard 5-year surveillance window is insufficient. These findings support extending ophthalmologic surveillance beyond the standard 5-year window. We propose, based on our retrospective institutional data, a 10-year functional survivorship framework.

## Simple Summary

Orbital rhabdomyosarcoma is a rare childhood cancer with high survival rates. However, treatment can lead to long-term eye problems. We studied 22 children over 23 years at a major cancer center in Jordan to understand these late effects. We found that most patients survived (84%), but half developed cataracts, sometimes nearly eight years after treatment. We also observed that if the tumor progressed during initial chemotherapy, it may be an early warning sign for poor outcomes, though this finding is based on a small number of patients and needs further study. Based on these findings, we suggest that children with this cancer be followed by an ophthalmologist for at least 10 years, rather than the standard five, to catch and treat late-occurring complications.

## 1. Introduction

Rhabdomyosarcoma (RMS) is the most common soft tissue sarcoma of childhood, and the orbit is one of its most frequent primary sites in the head and neck region [1,2]. Orbital RMS accounts for approximately 10% of all pediatric RMS and is the most common primary malignant orbital tumor in children, with a mean age of onset of 5–7 years and a slight male predominance [3,4]. Unlike most other RMS sites, the orbit produces early and visible signs—proptosis, eyelid swelling, or chemosis—that typically lead to diagnosis before systemic dissemination, contributing to the favorable prognosis of this anatomical location [5,6].

Before the multimodality treatment era, orbital exenteration achieved cure in fewer than 30% of patients [7]. The sequential Intergroup Rhabdomyosarcoma Study (IRS) protocols established that a combination of vincristine, actinomycin-D, and cyclophosphamide (VAC) chemotherapy and external-beam radiation therapy (EBRT) could achieve orbital preservation rates above 90% with 5-year overall survival exceeding 85–95% for localized disease [8,9,10,11,12,13,14,15]. This therapeutic success, however, carries a substantial burden of late ophthalmologic toxicity: radiation-induced cataracts develop in 55–82% of irradiated patients in historical series, decreased visual acuity affects the majority of long-term survivors, and additional sequelae including ptosis, orbital hypoplasia, kerato-conjunctivitis, and retinopathy are common [9,16,17,18].

Current practice has shifted toward minimizing radiation exposure in favorable responders. European groups have explored chemotherapy-only approaches for selected non-parameningeal orbital cases, and the Institute Curie experience demonstrated that radiation can be deferred in patients achieving complete or excellent clinical and radiologic responses to induction chemotherapy without compromising survival [19]. Systematic documentation of Magnetic resonance imaging (MRI) response prior to local control has therefore become an important clinical tool for guiding treatment decisions and potentially quantifying response as a surrogate endpoint in future trials [20,21,22].

Despite these advances, data from low- and middle-income countries (LMICs), where the majority of the world’s children with cancer live and where referral patterns, resource constraints, and treatment protocols may differ substantially from cooperative-group contexts, remain sparse. King Hussein Cancer Center (KHCC) is a dedicated tertiary oncology center that treats most pediatric malignancies in Jordan, providing a geographically complete cohort. Here we report the clinical characteristics, MRI chemotherapy response, treatment outcomes, and ophthalmologic late effects of children with primary orbital RMS treated at KHCC over 23 years.

## 2. Materials and Methods

### 2.1. Study Design and Patients

We conducted a retrospective single-institution analysis of all patients younger than 18 years diagnosed with primary orbital RMS and treated at KHCC between January 2002 and February 2025. Cases were identified from the institutional cancer registry. Patients with parameningeal extension into orbital structures and those with orbital involvement as a component of disseminated disease were excluded. Patients were excluded if they presented solely for radiation therapy, initiated chemotherapy at an outside facility, or did not complete their treatment course at our institution.

### 2.2. Data Collection

Clinical and demographic data extracted from medical records included age at diagnosis, sex, presenting symptoms (specifically proptosis), primary tumor size (≤5 cm vs. >5 cm), regional lymph node status by imaging, PAX-FOXO1 fusion status by fluorescence in situ hybridization (FISH), Children’s Oncology Group (COG) risk-group assignment. Additionally, treatment variables were recorded, including initial surgical procedure, radiation dose, and MRI response assessment before local control. Response was classified from the interpreting radiologist’s clinical report as CR (complete disappearance of tumor on MRI), PR (>30% reduction in longest diameter), SD/MR (minor response or stable disease, <30% reduction), or PD (progressive disease, >30% increase). Survival data included vital status, events, event-free survival (EFS) and overall survival (OS) durations in months from diagnosis.

Ophthalmologic data were obtained from routine clinical follow-up visits with the institutional ophthalmology service and were not collected as a standardized prospective research protocol. Visual acuity assessments were performed by ophthalmologists at each visit using age-appropriate methods. In infants (<1 year), visual function was assessed qualitatively based on light perception and ability to fix and follow. In young children (<3 years), visual acuity was evaluated using LEA symbols. In children ≥ 3 years, visual acuity was measured using standard charts and recorded in decimal notation. The timing and completeness of ophthalmologic evaluations varied across the 23-year study period, reflecting the evolution of institutional practice. Visual acuity data were available for 20 of 22 patients; two patients had incomplete ophthalmologic records and were excluded from the relevant outcome analyses. Late ophthalmologic effects were abstracted from clinical charts rather than collected through a dedicated survivorship registry. Variables extracted included visual acuity (VA) in the affected eye at last assessment, presence and date of cataract formation, and other documented ocular findings. For the purposes of this study, ‘Good Visual Acuity’ was defined as a Snellen fraction of ≥20/40 (decimal ≥ 0.5 or logMAR ≤ 0.3), which represents the threshold for functional independence in most pediatric daily activities, moderate (VA 0.1–<0.5), or severe impairment (counting fingers, hand motion, or no light perception/blind).

### 2.3. Treatment Approach

All patients with orbital RMS were treated with VAC chemotherapy with low-dose cyclophosphamide (1.2 gm/m^2^) throughout the study period. Local control was primarily through radiotherapy in all patients after induction chemotherapy; the dose of radiotherapy was 45 Gy for all patients treated before 2018, thereafter, the dose was tailored according to response to chemotherapy, with 45 Gy to those achieving CR and 50.4 Gy for those achieving less than CR, if feasible. Radiation therapy was delivered using conformal techniques, predominantly utilizing 3D conformal radiation therapy (3D-CRT) or intensity-modulated radiation therapy (IMRT). Lens-sparing approaches were prioritized where anatomically feasible to minimize anterior segment toxicity. While primary surgical intervention was largely restricted to incisional biopsy, a few patients underwent attempted resection prior to institutional referral.

MRI of the orbit was performed routinely after induction chemotherapy (typically 4–6 cycles) and prior to the start of local control. Response was classified from radiology reports as CR, PR, SD/MR, or PD.

### 2.4. Statistical Analysis

Patient and tumor characteristics are summarized as frequencies and proportions for categorical variables and as median with IQR for continuous variables. Event Free Survival (EFS) was defined as the time from diagnosis to the first relapse, progression, or death from any cause, with censoring at last contact. Overall Survival (OS) was the time from diagnosis to death from any cause. Both were estimated by the Kaplan–Meier method. Given the descriptive nature of this analysis and small sample size, no formal hypothesis tests were applied to survival comparisons. All analyses were performed in R version 4.4.3.

## 3. Results

### 3.1. Patient and Tumor Characteristics

Twenty-two patients were treated for primary orbital RMS between 2002 and 2025. Median age at diagnosis was 5.6 years (IQR 4.1–8.5). There were 13 (59%) males (Male-to-Female ratio 1.4:1). Proptosis was documented at presentation in 7 patients (32%). All patients had localized disease at diagnosis with no distant metastases. Table 1 presents the full patient and tumor characteristics.

**Figure 1:**
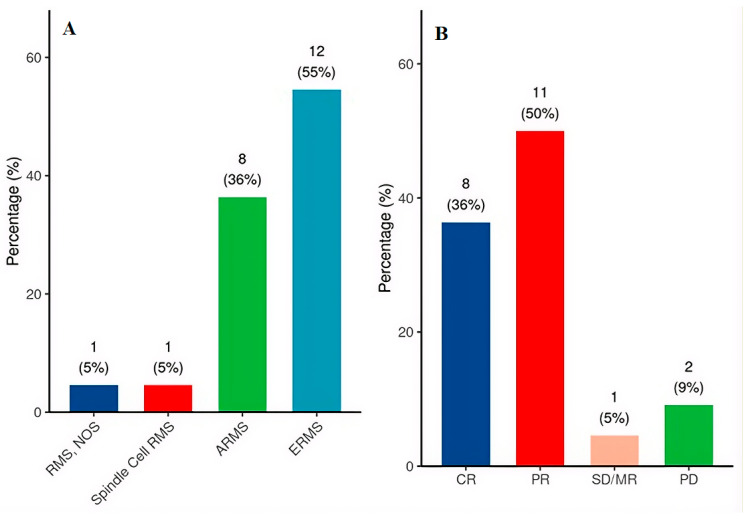
(**A**) Histological subtype distribution (*N* = 22). (**B**) MRI response classification before local control (*N* = 22). CR = complete response; PR = partial response; SD/MR = stable/minor response; PD = progressive disease; ERMS = embryonal rhabdomyosarcoma; ARMS = alveolar rhabdomyosarcoma; RMS, NOS = Rhabdomyosarcoma, not otherwise specified.

Embryonal RMS (ERMS) was the most common histological subtype (12/22 (55%)), followed by alveolar (ARMS) (8/22 (36%)) (Figure 1A). Tumors exceeded 5 cm in 13 patients (59%), and regional lymph node involvement by imaging was present in two patients. PAX-FOXO1 FISH testing was performed in 9 of 22 patients (41%); one was fusion-positive (ARMS histology). Risk stratification placed 17 patients (77%) in the COG low-risk group and 5 (23%) in the intermediate-risk group; no patient met criteria for high-risk disease.

### 3.2. Treatment

All 22 patients received VAC chemotherapy with low-dose cyclophosphamide. EBRT was given to 20 patients (91%); 16 (80% of irradiated patients) received 45 Gy and four received 50.4 Gy (median 45 Gy). Of the 20 irradiated patients, 11 were treated before 2018 (10 receiving 45 Gy and one 50.4 Gy) and 9 from 2018 onward (6 receiving 45 Gy and 3 receiving 50.4 Gy per the response-adapted protocol). The cataract rate was numerically higher in the pre-2018 group (8/11 [73%] vs. 2/9 [22%]); however, this difference is heavily confounded by the substantially longer ophthalmologic follow-up in the earlier cohort (median 95.9 vs. 21.2 months). Given that the median cataract latency in this series was 39.1 months and the maximum reached 95.1 months, many post-2018 patients have not yet reached the timeframe during which cataracts typically manifest. Similarly, good visual acuity was more frequent in the post-2018 group (67% vs. 44%), but the small subgroup sizes and differential follow-up preclude formal comparison. No difference in survival was apparent between eras, though the post-2018 cohort remains early in follow-up. Two patients did not receive radiation because their tumors progressed despite neoadjuvant chemotherapy; following this treatment-refractory course, both patients succumbed to disease. Surgery was performed in two patients (9%), both of whom underwent an attempted excision at diagnosis before referral to KHCC; one had unknown surgical resection margin and one had residual microscopic disease. All remaining patients had a diagnostic biopsy only.

### 3.3. MRI Response Before Local Control

MRI response data were available for all patients (100%). Of these, eight patients (36%) achieved CR, 11 (50%) achieved PR, one (5%) had SD/MR and two patients (9%) experienced PD (Figure 1B). Objective response (CR or PR) was associated with excellent salvage ability; only two fatal events occurred among the 19 patients (10.5%) who achieved CR or PR. In contrast, mortality was exclusively concentrated in the non-responder group. While the single patient with SD/MR experienced an EFS event but remained alive at last follow-up, both patients with PD succumbed to their disease. In the CR cohort, only one patient—a two-month-old infant with low-risk ERMS—progressed rapidly following an initial response (EFS = 5.6 months) and passed away. Similarly, only one event occurred in the PR group, involving a 4.5-year-old female with ARMS who died following a local recurrence 32.5 months after diagnosis. The distribution of MRI responses and their correlation with patient outcomes are detailed in Table 2.

### 3.4. Survival Outcomes

Four patients (4/22 (18%)) died and five (5/22 (23%)) experienced an event during follow-up. Among the 5 events, four were local recurrences and one was a distant brain metastasis. Among the 18 patients alive at last contact, the median follow-up was 85.9 months.

**Figure 2:**
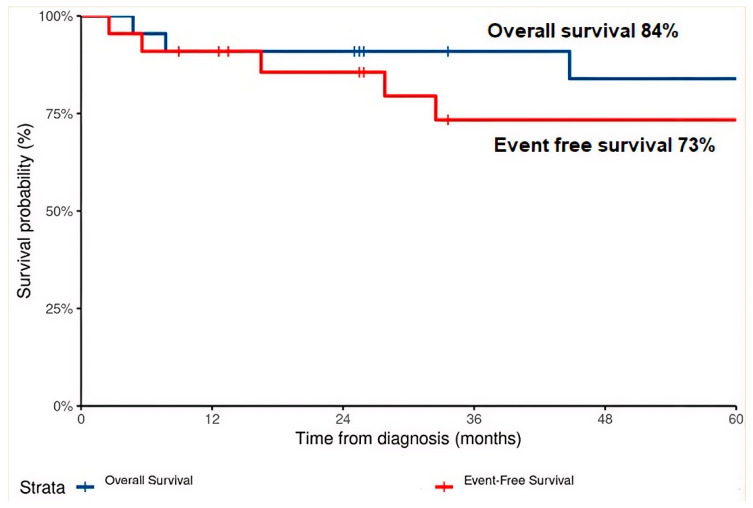
Kaplan–Meier estimates of overall survival (OS) and event-free survival (EFS) for all 22 patients.

At a median follow-up of 85.9 months for surviving patients, median OS and median EFS were not reached. The estimated 5-year EFS and OS rates were 73% and 84% respectively (Figure 2). All four deaths occurred in patients with ERMS histology; no ARMS patient died, though one experienced an event (local recurrence, subsequently alive).

### 3.5. Ophthalmologic Outcomes

The median time from diagnosis to last ophthalmologic assessment among the 20 evaluable patients was 103.6 months (range 0.5–271.8). Ophthalmologic evaluations were performed as part of routine clinical follow-up rather than at standardized time points; therefore, the timing of assessments varied among patients.

**Figure 3:**
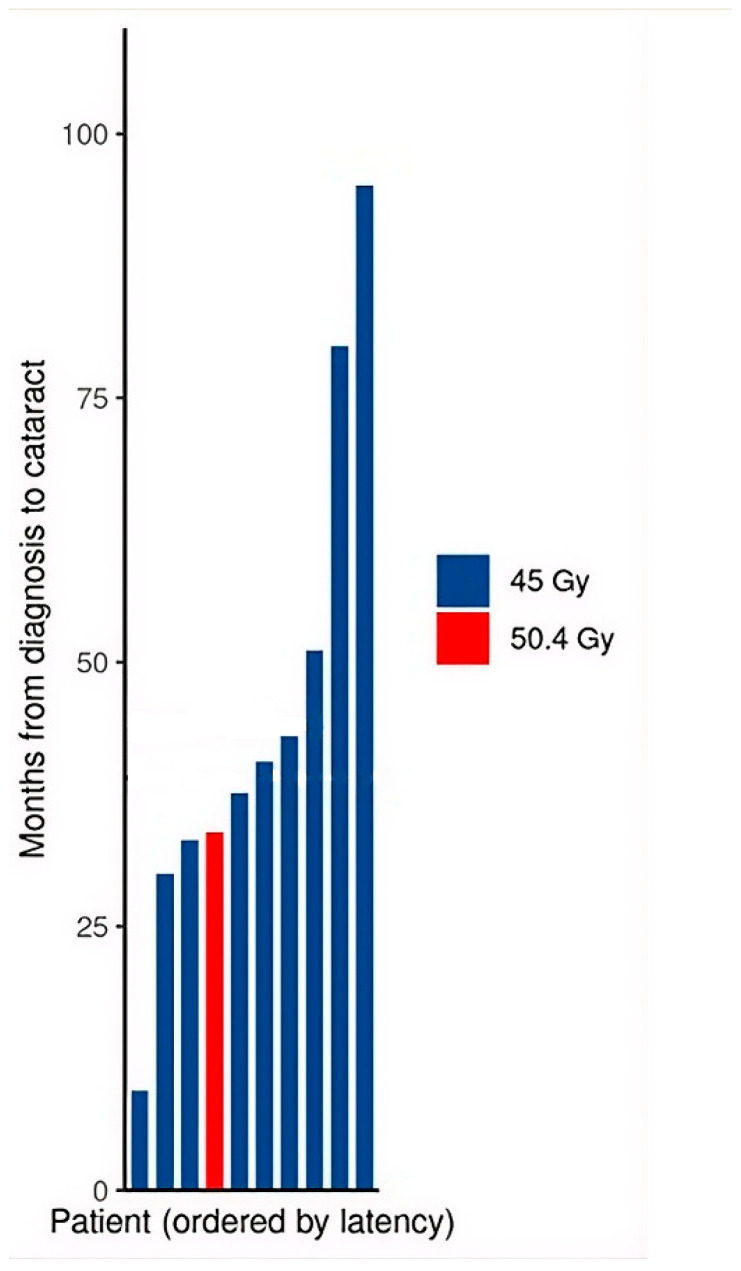
Ophthalmologic outcomes. Time from diagnosis to cataract formation for the 10 patients with a recorded cataract date, colored by radiation dose.

Cataracts developed in 10 of 22 patients (45%), corresponding to 10/20 (50%) among irradiated patients. The median time from diagnosis to cataract formation was 39.1 months (range 9.4–95.1), with all cases occurring within 8 years of diagnosis (Figure 3).

Visual acuity (VA) at last assessment was available for all 20 irradiated patients. Of these, 12 (60%) had good VA, 5 (25%) had moderate impairment, and 3 (15%) had severe functional impairment. Other documented ocular findings included ptosis in five patients (requiring surgical correction in three), corneal scarring/opacity in seven, and enophthalmos in one. Ophthalmologic outcomes are summarized in Table 3.

The etiology of non-cataract ocular findings varied; ptosis (*n* = 5) was predominantly associated with initial tumor-related mechanical disruption and surgical manipulation at diagnosis. In contrast, corneal opacities (*n* = 7) were linked to severe proptosis and exposure keratopathy at presentation, while enophthalmos appeared as a late sequela of radiotherapy.

## 4. Discussion

This series of 22 children with primary orbital RMS treated at KHCC over 23 years is the largest institutional report from Jordan and includes, to our knowledge, the first systematic MRI chemotherapy response data from a Middle Eastern pediatric cohort. The cohort reflects the favorable epidemiology of orbital RMS: all patients presented with localized disease, the majority were low-risk, and survival outcomes—while not captured through a formal cooperative group protocol—are consistent with international series. The orbital site carries a relatively favorable prognosis partly because ERMS predominates and because orbital tumors tend to be detected early at smaller sizes. In our cohort, 55% (12/22) of patients had ERMS, consistent with published data.

Unlike parameningeal, extremity, or bladder/prostate primaries, orbital RMS presents early before regional nodal involvement or distant dissemination develops, owing to the paucity of orbital lymphatics [1,23]. None of the patients in our study presented with distant metastasis. However, imaging revealed regional lymph node involvement in two patients (9%), one of which was pathologically proven.

The availability of systematic, pre-local-control MRI response data is the principal new contribution of this updated series. Following induction chemotherapy, 8 (36%) achieved CR and 11 (50%) PR. Our data suggest that the response to induction therapy could carry a stratified prognostic value. The single patient with SD/MR experienced a local recurrence that was successfully salvaged. Conversely, PD on induction MRI was associated with mortality in both affected patients (2/2). While limited by the small sample size and therefore strictly hypothesis-generating, these findings raise the possibility that frank progression identifies a particularly high-risk subgroup.

This distinction is noteworthy; while any lack of regression warrants concern, frank progression during VAC chemotherapy could identify an ultra-high-risk phenotype that may be refractory to standard salvage protocols even in a traditionally ‘favorable’ site like the orbit. Such patients may benefit from immediate molecular profiling and a rapid shift toward intensified or novel therapeutic strategies. However, as this subgroup included only two patients, larger prospective studies are necessary to validate the predictive value of early radiographic progression in orbital RMS.

Our results align with larger studies emphasizing the predictive power of radiographic response. The Institute Curie series demonstrated that CR or near-CR on MRI could identify patients in whom radiation therapy can be safely avoided, reporting no excess events in the 17 of 95 patients with orbital RMS spared radiation after an excellent response [19]. The COG ARST0331 trial established that pre-radiotherapy response strongly predicts local control; patients achieving a CR at week 12 had zero local recurrences following 45 Gy of radiation, whereas those with a sub-optimal response faced a significantly higher recurrence rate (6 of 38) [20]. This highlights that while 45 Gy is highly effective for complete responders, it is inadequate for partial responders, underscoring the need for intensified local therapies to ensure durable control. Therefore, prospective documentation of MRI response in all future patients, using standardized RECIST-based criteria, should be prioritized to build the evidence base for response-adapted local therapy.

The cataract rate of 45% (10/22 overall, 50% of irradiated patients) is lower than the 55–82% rates reported in historical IRS series using higher doses and older radiotherapy techniques, yet it remains a substantial burden [9,16,24]. The median latency of 39.1 months from diagnosis to cataract formation has direct implication for follow-up protocols. Because the maximum latency for cataract development in our cohort reached 95.1 months (nearly eight years), a standard discharge from specialized care at the five-year mark would fail to capture a significant portion of forthcoming ocular complications. This concern is underscored by our era-based comparison: the apparently lower cataract rate in patients treated from 2018 onward (22% vs. 73% in the pre-2018 group) is largely attributable to the shorter ophthalmologic follow-up in the more recent cohort (median 21.2 vs. 95.9 months), rather than to a true difference in cataract incidence. This observation reinforces the need for extended surveillance, as late-onset cataracts may not yet have manifested in patients with shorter follow-up.

While current COG Long-Term Follow-Up Guidelines (Version 6.0) recommend annual ophthalmologic screening for survivors exposed to orbital radiation to monitor for cataracts and retinal changes [25], our data reveal a critical ‘survivorship gap’. Given the high survival rates and the nearly 8-year latency window, a standard 5-year discharge from ophthalmologic follow-up is clinically insufficient. Based on our single-institution retrospective experience, we propose an extended Orbital RMS Survivorship Protocol. While this recommendation requires prospective validation, the prolonged cataract latency observed in our cohort supports extending ophthalmologic surveillance beyond the conventional 5-year window. This extended window is essential for timely secondary interventions, such as modern cataract extraction, as 60% of our cohort retained good visual acuity (VA ≥ 0.5) at last assessment.

This survivorship gap is often compounded by the transition of care. Once patients are discharged from acute oncology follow-up, they often return to primary care settings where specialized tools, such as slit-lamp biomicroscopy, are unavailable.

At the last assessment, 60% of the evaluable patients retained good VA (≥20/40). Our functional outcomes compare favorably with landmark international series, particularly regarding the balance between disease control and late toxicity [26]. While historical studies like the IRS-III reported a high 5-year OS of 91%, they also recorded a significantly heavier burden of ocular morbidity, including an 82% cataract rate and decreased visual acuity in 70% of survivors [9]. The Wills Eye series found that 43% of patients suffered from severe visual impairment (VA ≤ 20/200) [3].

In contrast, our cohort achieved a comparable 5-year OS of 84% with a markedly lower severe visual impairment rate (15% among irradiated patients). This improvement may, in part, reflect the use of a lower median radiation dose (45 Gy vs. historically >50 Gy), contemporary conformal EBRT techniques, and modern cataract extraction [24,27,28,29,30]. Direct comparisons with historical series should be interpreted cautiously; differences in treatment era, radiation doses and techniques, patient selection, and follow-up duration may contribute to the observed differences in ocular toxicity rates. While visual loss remains a substantial long-term risk, our outcomes demonstrate that excellent results for orbital RMS are achievable in a tertiary LMIC setting. By achieving high survival rates that align with international benchmarks while minimizing severe morbidity, this experience supports the feasibility of achieving favorable outcomes in resource-stratified settings that prioritizes both oncologic cure and functional preservation.

Although our cohort size (*N* = 22) reflects the rarity of this primary site, the 23-year duration provides a unique longitudinal perspective on late effects that is often missing from larger, shorter-term studies. Furthermore, achieving an 84% 5-year OS in a tertiary LMIC center demonstrates that high-quality, resource-stratified care—utilizing low-dose cyclophosphamide and conformal EBRT—can yield results that broadly consistent with COG and international benchmarks while maintaining a low rate of severe visual impairment.

This retrospective, single-institution analysis of a small cohort has several inherent limitations. MRI responses were abstracted from clinical radiology reports rather than being centrally reviewed by a single radiologist using standardized RECIST criteria. This approach may introduce inter-observer classification variability, and the associations between MRI response categories and clinical outcomes should therefore be interpreted with this methodological limitation. Similarly, our ophthalmologic findings are limited by the retrospective nature of the data; visual acuity assessments were pulled from routine follow-up visits rather than a standardized survivorship protocol. This lack of uniformity in the timing of assessments may introduce variability in the recorded onset of late effects. Additionally, the small number of overall events in this orbital series (*n* = 5) precludes multivariable analysis of prognostic factors. Furthermore, while the total prescribed orbital radiation doses were documented, detailed dosimetric parameters for the lens (e.g., maximum or mean lens dose, dose-volume histograms) were not systematically retrievable for historical cases treated over the 23-year study period. This limits our ability to perform a dose–response analysis for cataract formation and to correlate specific dose-volume metrics with the observed variation in cataract latency. Finally, while we observed a lower cataract rate in patients treated from 2018 onward compared with the earlier cohort (22% vs. 73%), the substantially shorter ophthalmologic follow-up in the more recent group (median 21.2 vs. 95.9 months) is a major confounder, and the true long-term ocular toxicity burden in these patients remains to be determined.

### Proposed Institutional Survivorship Recommendation

Given a maximum cataract latency of 95 months in our cohort, a standard 5-year discharge from ophthalmologic follow-up is clinically insufficient. We propose a 10-Year Orbital RMS Survivorship Protocol to address the ‘survivorship gap’:

- Oncologic Phase (Years 1–5): Combined oncology and ophthalmology surveillance focused on local control.
- Functional Preservation Phase (Years 6–10): Annual ophthalmologic screening focused on late-onset cataracts and corneal morbidity.
- The Functional Handover: A formal transfer of care to a community ophthalmologist at year 5, ensuring longitudinal slit-lamp examinations continue through year 10. This ensures that treatable visual morbidity is not neglected in a population that otherwise achieves excellent survival.

## 5. Conclusions

Orbital RMS at our institution presents primarily as localized, low-risk disease, with survival outcomes that align with international cooperative group benchmarks. Our data suggest that MRI response may serve as a guide for future response-adapted therapies and a potentially important early prognostic signal, with induction PD possibly identifying patients at higher risk of treatment failure, though this finding is based on only two patients and requires validation in larger cohorts.

Regarding late effects, the 50% cataract rate and prolonged latency among irradiated patients confirm that standard survivorship timelines are insufficient. Because a proportion of radiation-induced ocular toxicities in our study emerged after the standard 5-year surveillance window, extending up to 95 months post-diagnosis, discharging patients from ophthalmologic follow-up at 5 years may result in missed, treatable visual morbidity in a population that otherwise achieves excellent long-term survival. Based on these retrospective single-institution observations, we recommend that centers consider extending annual ophthalmologic screening, including slit-lamp examination and visual acuity testing, to at least 10 years post-diagnosis; however, this recommendation requires validation in prospective, multi-institutional studies.

## Figures and Tables

**Table 1:** Patient and Tumor Characteristics (*N* = 22).

Variable	*N* = 22 ^1^
Age at diagnosis (years)	5.6 [4.1–8.5]; range 0.2–17.0
Age group	
<5 years	9 (41%)
5–<10 years	9 (41%)
10–<15 years	3 (14%)
≥15 years	1 (4.5%)
Sex	
Female	9 (41%)
Male	13 (59%)
Proptosis at presentation	7 (32%)
Histological subtype	
ARMS	8 (36%)
ERMS	12 (55%)
RMS, NOS	1 (4.5%)
Spindle Cell RMS	1 (4.5%)
Tumor size	
≤5 cm	9 (41%)
>5 cm	13 (59%)
Regional nodes positive (imaging)	2 (9.1%)
COG risk group	
Low	17 (77%)
Intermediate	5 (23%)
High	0 (0%)
PAX-FOXO1 fusion (FISH)	
Negative	8 (89%)
Positive	1 (11%)
Not tested	13

^1^ Median [25–75%]; range Minimum–Maximum; *n* (%). Abbreviations: ARMS, alveolar rhabdomyosarcoma; ERMS, embryonal rhabdomyosarcoma; RMS, rhabdomyosarcoma; NOS, not otherwise specified; FISH, fluorescence in situ hybridization; COG, Children’s Oncology Group.

**Table 2:** Outcomes by MRI Response Category Before Local Control (*N* = 22).

MRI Response	*N*	Received EBRT (*N*)	Deaths	EFS Events	Median Follow-Up (Months)
CR	8	7	1	1	85.9
PR	11	11	1	1	72.6
SD/MR	1	1	0	1	25.0
PD	2	1	2	2	24.8

Abbreviations: MRI, Magnetic resonance imaging; EBRT, external beam radiotherapy; CR, complete response; PR, partial response; SD/MR, stable disease/minimal response; PD, progressive disease.

**Table 3:** Ophthalmologic Outcomes in Irradiated Patients.

Variable	Yes, *N* = 20 *	VA Range (Snellen)	VA Range (logMAR)
Cataracts	10 (50%)	-	
Visual acuity at last assessment			
Good vision	12 (60%)	≥20/40	≤0.3
Moderate impairment	5 (25%)	20/50 to 20/200	0.4 to 1.0
Severe impairment	3 (15%)	<20/200 to blind	>1.0
Ptosis	5 (25%)		
Corneal opacity/scarring	7 (35%)		

Abbreviations: VA, visual acuity. * Two patients who did not receive EBRT are excluded. Visual acuity data were available for all 20 irradiated patients.

## Data Availability

De-identified data are available upon reasonable request from the corresponding author.
